# Features of the wound healing process in mice during photodynamic therapy with fotoditazin and methylene blue in the presence of amphiphilic polymers and sodium alginate

**DOI:** 10.3389/fchem.2026.1852721

**Published:** 2026-07-24

**Authors:** Valeriya Kardumyan, Rehanguli Aimaier, Alexey Fayzullin, Anastasia Kuryanova, Ru-Lin Huang, Qingfeng Li, Yana Khristidis, Peter Timashev, Anna Solovieva

**Affiliations:** 1 N N Semenov Federal Research Center for Chemical Physics, Russian Academy of Sciences, Moscow, Russia; 2 Department of Plastic and Reconstructive Surgery, Shanghai Ninth People’s Hospital, Shanghai Jiao Tong University School of Medicine, Shanghai, China; 3 Shanghai Institute for Plastic and Reconstructive Surgery, Shanghai, China; 4 Institute for Regenerative Medicine, Sechenov University, Moscow, Russia

**Keywords:** photodynamic therapy, photosensitizers, photostability, polymers, singlet oxygen, wound healing

## Abstract

**Introduction:**

The development of photosensitizers with high tissue penetration and strong antimicrobial activity against pathogenic microorganisms is at the forefront of antimicrobial photodynamic therapy research. Reducing the aggregation of photosensitizers (PS) in aqueous solutions through the addition of amphiphilic polymers can enhance their ability to generate reactive oxygen species (ROS) and, consequently, improve the efficacy of photodynamic treatment. Previously, we demonstrated that amphiphilic polymers increase the singlet oxygen generation activity of porphyrin photosensitizers and certain dyes by promoting the disaggregation of hydrophobic PS molecules.

**Results and Discussion:**

In the present study, we performed a comparative investigation of photodynamic treatment of model wounds in mice using methylene blue dye and Fotoditazin as PS, both in the presence and absence of the amphiphilic polymer Pluronic F108. In addition, sodium alginate was incorporated into the photosensitizing systems to provide an additional antibacterial effect on the wound.

**Conclusion:**

These findings provide a theoretical basis for the application of photosensitizer–polymer systems containing sodium alginate in antimicrobial photodynamic therapy for difficult-to-treat wounds.

## Graphical Abstract

**Figure:**
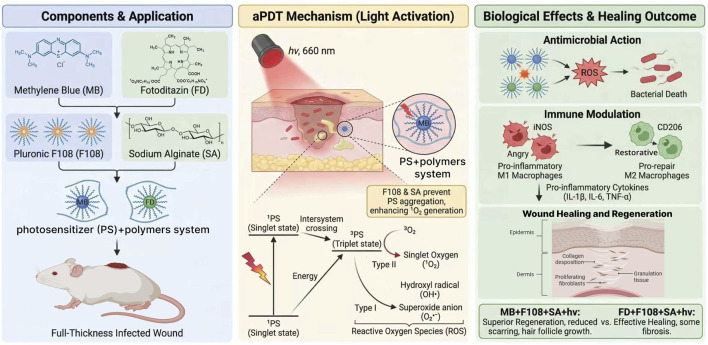

## 1 Introduction

**SCHEME 1:**
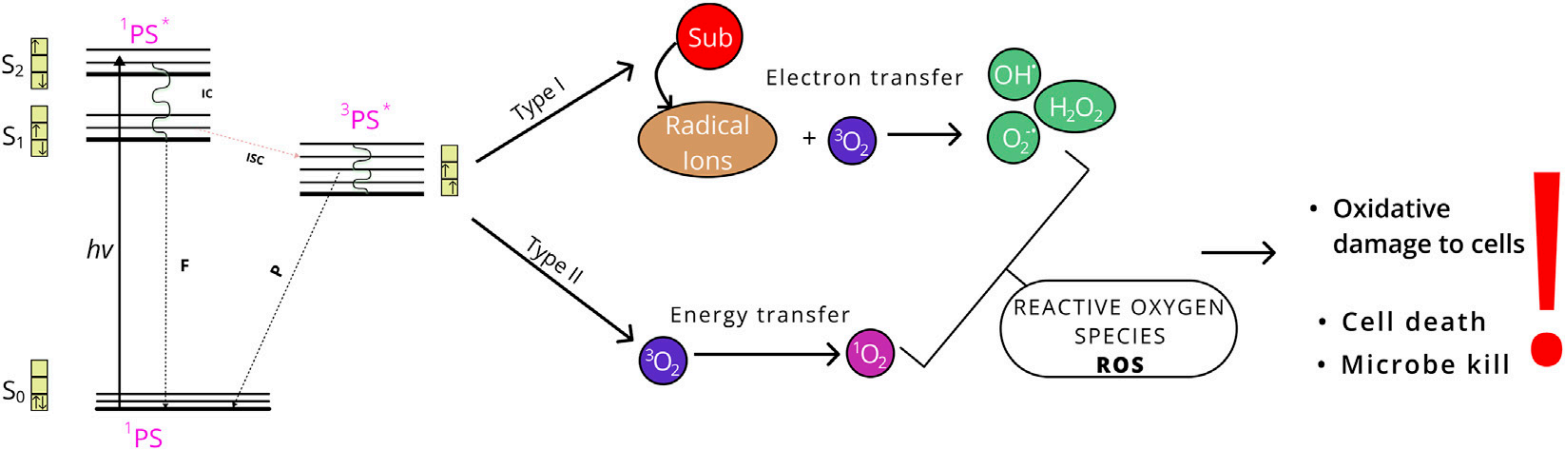
Photosensitized oxidation reactions involving the type I and type II mechanisms.

Photodynamic therapy (PDT), a minimally invasive tumor treatment method developed in the mid- 20th century and is associated with laser technologies in medicine, is now a recognized therapeutic strategy in oncology (Correia et al., 2021; Gunaydin et al., 2021). PDT is based on the oxidative destruction of pathological cells by reactive oxygen species (ROS), generated by excited photosensitizers (PS) that selectively accumulate in cancer cells. Subsequent studies demonstrated that in addition to cancer cells, many biological organisms (bacteria, viruses, fungi) can also accumulate PS, rendering them susceptible to low-intensity laser radiation of the appropriate wavelength (Gilaberte et al., 2021). Owing to the growth of antibiotic resistance and the need for alternative antimicrobial strategies, PDT is increasingly being used in clinical practice to treat localized infections (Dai et al., 2009; Jori et al., 2006). In particular, PDT has been applied in the treatment of infectious and inflammatory diseases (Di Bartolomeo et al., 2022; Krupka et al., 2021), as well as in purulent surgery (Demidova-Rice et al., 2012; Domka et al., 2024; Guo et al., 2024; Peplow et al., 2012; Radjabov et al., 2017), otolaryngology (Domka et al., 2024), dentistry (Gholami et al., 2023; Hirose et al., 2021; Joseph et al., 2017; Meimandi et al., 2017), and ophthalmology (Mitropoulos et al., 2014). Importantly, photodynamic treatment is effective against multidrug-resistant bacterial strains (Hu et al., 2018; Morales-de-Echegaray et al., 2018; Sun et al., 2020). It should also be noted that, unlike antibiotics, which act on specific cellular targets such as the cell wall, cytoplasmic membrane, DNA replication, transcription, or protein synthesis, ROS induce non-specific oxidative damage to multiple cellular components thereby substantially reducing the likelihood of microorganisms developing resistance to repeated photodynamic treatments (Almeida, 2020; Di et al., 2019; Hua et al., 2024). Antimicrobial photodynamic therapy (aPDT) requires three essential components: a photosensitizer, light of an appropriate wavelength corresponding to the absorption spectrum of the photosensitizer, and molecular oxygen. Among these components, the photosensitizer plays a central role in determining the efficiency of the photodynamic process. Upon illumination, PS is excited to a triplet excited state and promotes the formation of reactive oxygen species (ROS) via two mechanisms (Scheme 1): (1) a type I mechanism in which an electron is transferred from PS to an adjacent oxygen atom, forming highly reactive radical species including hydroxyl radicals and superoxide anions; and (2) a type II mechanism in which singlet oxygen (^1^O_2_) is generated through energy transfer between PS and an adjacent oxygen atom (Di et al., 2019; Guo et al., 2025). The efficacy of PDT is largely determined by the ability of the photosensitizer to generate reactive oxygen species (ROS), particularly singlet oxygen (^1^O_2_), as well as by its photostability and aqueous solubility (Hamblin, 2017; Yogo et al., 2005).

Some of the most widely used and relatively low-toxic photosensitizers in antimicrobial photodynamic therapy (aPDT) are porphyrin compounds, particularly chlorin e6 and its derivatives (Semenov et al., 2023). In recent years, anthracene dyes including eosin, riboflavin, toluidine blue, rose bengal, and methylene blue (MB), have been investigated as PS for aPDT (Almenara-Blasco et al., 2024; Nagai De Lima et al., 2026). Compared to porphyrins, these dyes are generally more photostable and readily available, and possess both photosensitizing and intrinsic antibacterial properties (Usacheva et al., 2003; Zharova et al., 2016). In this study, we performed a comparative investigation of photodynamic treatment of model wounds in laboratory mice using the diglutamine salt of chlorin e6 (Fotoditazin) and the cationic dye methylene blue. Fotoditazin is an effective photosensitizer with a high singlet oxygen ^1^O_2_ quantum yield (ФΔ ∼ 0.6-0.7) (Fontana et al., 2022; Kustov et al., 2021). As a photosensitizer, MB is less efficient than Fotoditazin in generating reactive oxygen species and exhibits a lower singlet oxygen quantum yield (ΦΔ ≈ 0.5–0.6). To enhance ROS generation, we employed complexes of the photosensitizer with an amphiphilic polymer Pluronic F108. Previous studies have shown, that Pluronic F108 reduces nonspecific toxicity and modulates the aggregation state of photosensitizers (Kuryanova et al., 2023; Solovieva et al., 2020). For example, mitomycin C in combination with Pluronic exhibits enhanced and prolonged therapeutic activity while demonstrating lower toxicity than the free drug (Isakau et al., 2008; Yu et al., 2021). We previously demonstrated that increasing the concentration of FD or MB in a model reaction of tryptophan photooxidation in water decreases the effective rate constant (keff) of the process, which is associated with dye aggregation under these conditions (Kuryanova et al., 2023; Shershnev et al., 2025; Solovieva et al., 2020). In contrast, in the presence of amphiphilic polymers (AP), such as PVP, PVA, F127, and others, no decrease in keff is observed with increasing photosensitizer concentration (Solov’eva et al., 2019). Moreover, the photocatalytic activity of water-soluble porphyrins in the tryptophan oxidation reaction increases in the presence of AP owing to the formation of weakly bound intermolecular PS-AP complexes and the disaggregation of photosensitizer molecules (Kuryanova et al., 2023; Solovieva et al., 2020). To further improve the efficacy of aPDT, biologically active compounds (BACs) such as growth factors, bactericidal agents, and proteolytic enzymes, can be administered alongside photodynamic treatment to provide additional therapeutic benefits during wound healing. In this study, sodium alginate (SA) obtained by alkaline extraction from brown algae and intrinsic bactericidal properties, was used as a BAC. SA exhibits hemostatic properties and promotes the healing of ulcerative lesions of the gastric and intestinal mucosa (Chin et al., 2008).

The aim of this study was to investigate the relationship between singlet oxygen generation, the photostability of structurally different photosensitizers (Fotoditazin and methylene blue), and the efficacy of antimicrobial photodynamic therapy (aPDT) of model wounds in laboratory animals. Although photosensitizers often exhibit excellent photophysical and photochemical properties *in vitro*, their actual efficacy in the complex *in vivo* environment, particularly in wound healing models, requires rigorous validation in animal studies. To this end, we established a full-thickness skin defect model in mice and evaluated the therapeutic efficacy of polymer–photosensitizer systems based on methylene blue and Fotoditazin *in vivo*. Through an integrated evaluation that included macroscopic assessment of wound healing and microscopic histological analysis, we systematically investigated how these photosensitizer systems modulate the inflammatory response and ultimately promote high-quality tissue regeneration. These *in vivo* findings provide an empirical basis for elucidating the mechanisms of aPDT-mediated wound healing and may facilitate the development of more effective therapeutic strategies.

## 2 Materials and methods

### 2.1 Reagents

Methylene blue (Unisource, Chimmed, Russia), a cationic phenothiazine dye (3,7-bis(dimethylamino)phenothiazine chloride, Mw 319.86 g/mol, ε(665 nm) = 64,800 M^−1^ x cm^−1^) and the medicinal product « Fotoditazin®» (concentrate for preparation of infusions solution, 5 mg/mL, DEKO, Russia) were used as a photosensitizer. Pluronic F108 (Mw 14.6 kDa, Fluka) was used as AP. Sodium alginate (SA), a polysaccharide with a molecular weight ranging from 10× 10^3^ to 600 × 10^3^ g mol^−1^(Neofroxx, Germany), was used as the biologically active compound (BAC). The structural formulas of these substances are shown in Supplementary Figure S1.

PS + F108, PS + SA, and PS + F108 + SA systems were prepared by mixing aqueous solutions of the corresponding components and stirring the mixtures magnetically for 15 min. The concentrations of the components used in spectroscopic studies are listed in Table 1.

**TABLE 1:** Сoncentration of components in the reaction solution.

Compound	Concentration
MB	2.5 × 10^−6^-1 × 10^−5^ М
FD	1 × 10^−6^–2.1 × 10^−5^ М
Pluronic F108	1 × 10^−4^ М
SA	0.00125 wt%

### 2.2 Spectral research

The electronic absorption spectra (EAS) of the PS in aqueous solutions, in the absence and presence of F108 and SA were recorded using a Cary 50 UV–Vis spectrophotometer (Varian, Australia). Irradiation of the reaction systems was performed using LED phototherapeutic apparatus (“Estus light”, JSC Geosoft Dent, Russia) emitting at wavelength of 660 nm and a power of 1000 mW.

The radiation dose was calculated according to the following equation:

$$D=\frac{P*t}{S}$$

where D is the radiation dose (J/cm^2^), P is the LED power (W), t is the irradiation time (sec) and S is the irradiation area (cm^2^).

The singlet oxygen luminescence spectra generated upon photoexcitation of the PS in the absence and presence of F108 and SA were recorded in D_2_O using a Horiba Fluoromax Plus spectrofluorimeter (Horiba-Jobin-Yvon, Palaiseau, France) equipped with an DSS-IGA020L IR detector (spectral range 800–1700 nm) and a TLP RG780 long-pass filter (Edmund Optics). The integrated area of the singlet oxygen luminescence peak at λ = 1276 nm was calculated using Origin 8.0 software.

### 2.3 Materials for PDT

The materials used in this work for PDT included Fotoditazin (chlorin e6 derivative, 2 mg/mL) and methylene blue (MB, 1 mg/mL), either alone or in combination with Pluronic F108 (20 mg/mL) and/or sodium alginate (5 mg/mL).

All photosensitizer—polymer systems were prepared by mixing the respective aqueous solutions to achieve the indicated final concentrations.

### 2.4 Experimental animal model

Specific-pathogen-free (SPF) male BALB/c mice (6 weeks old; weight, 22 ± 2 g; n = 32) were used in this study. A full-thickness skin excision (10 mm in diameter) was performed on the dorsal surface of each mouse under general anesthesia. To establish an infected wound model, a standardized suspension of *Staphylococcus aureus* (1 × 10^8^ CFU/mL) was applied topically to the wound. After 24 h, wound infection was confirmed by clinical signs and bacterial culture. The infected mice were then scored according to wound infection severity and assigned to nine intervention groups of 3–4 animals each (Table 2), ensuring comparable baseline infection severity among the groups. Animals were allocated using a stratified design based on infection severity scores. The animal experiments were approved by the Animal Care and Use Committee of Shanghai Ninth People’s Hospital, Shanghai Jiao Tong University School of Medicine (approval No. SH9H-2026-A240-SB).

**TABLE 2:** Groups allocation.

Group	Number of animals	Wound treatment
I(c)	3	No treatment
II	4	FD + hv
III	4	FD + F108 + hv
IV	4	FD + F108 + SA + hv
V	3	MB
VI	4	MB + hv
VII	3	MB + F108
VIII	3	MB + F108 + hv
IX	4	MB + F108 + SA + hv

### 2.5 Photosensitizer-mediated photodynamic therapy (PDT)

The mice were then anesthetized with inhaled isoflurane (3%–5%). The test solutions (total volume, 0.2 mL) were injected into the soft tissues of the wound bed using an insulin syringe at 2–3 equally spaced sites (approximately 0.05–0.1 mL per injection). Following administration of the test solutions, the wounds were incubated in the dark for 30 min by covering them with black cloth. During this period, mice from the same treatment group were housed together in the same cage.

In the non-irradiated group, after 30 min of dark incubation, the wounds were covered with sterile dressings, and the mice were returned to the SPF animal facility. In the remaining groups, PDT was performed using the “AFS” phototherapeutic apparatus (Polironic) equipped with an LED light source operating at a power of 1100 mW. The LED light source was mounted on a tripod, and the lamp head was positioned vertically at a distance of 1.5 cm from the wound surface. The radiation dose was 75 J/cm^2^, and the exposure time was 50 s. Two PDT sessions were performed on days 1 and 3 post-infection. Prior to the second PDT session, standardized wound photographs were obtained and repeat bacterial culture were performed.

### 2.6 Histological processing and staining

On day 13, all mice were humanely euthanized for histological analysis. During the 13-day observation period, wound morphology was documented by digital photography on days 0, 5, 9, and 13, and wound exudate samples were collected for bacterial culture and quantification of bacterial load.

Euthanasia was performed in accordance with the AVMA Guidelines for the Euthanasia of Animals (2020 Edition) published by the American Veterinary Medical Association. Carbon dioxide (CO_2_) inhalation was used for euthanasia. The procedure was conducted using a calibrated “Laboratory Animal Asphyxiator SMQ-II”; with the entire home containing the mice placed inside the chamber to minimize handling and transport stress. The CO_2_ displacement rate was set to 30%–70% of the chamber volume per minute to ensure rapid induction of unconsciousness while minimizing animal distress. Following gas exposure, death was confirmed in each animal by verifying the absence of vital signs. Before each procedure, the chamber was confirmed to be clean and free of residual gas to minimize animal stress.

Subsequently, full-thickness skin tissue, including the wound margin and surrounding normal skin, was carefully excised. The tissue samples were immediately immersed in 4% paraformaldehyde solution and fixed. After fixation, the samples were dehydrated through a graded ethanol series, cleared in xylene, and embedded in paraffin. The tissue blocks were sectioned into 5-μm-thick serial sections using a microtome and mounted on poly-L-lysine-coated glass slides. The slides were dried at 65 °C for 2 h prior to staining.

### 2.7 Hematoxylin and eosin (H&E) staining

The sections were deparaffinized in xylene and rehydrated through a graded ethanol series (100%, 95%, 85%, and 75%) to distilled water. The sections were stained with eosin solution for 2 min to visualize the cytoplasm and extracellular matrix, followed by nuclear staining with hematoxylin solution for 2 min. After rinsing under running water, differentiation was performed in 1% acid alcohol for 10 s, followed by bluing in running water for 10 min. Finally, the sections were dehydrated through a graded ethanol series, cleared in xylene, and mounted with neutral resin. The stained sections were examined using a slide scanner system.

### 2.8 Masson’s trichrome staining

Tissue sections were deparaffinized in xylene and rehydrated through a graded ethanol series to distilled water. The sections were mordanted and incubated overnight at room temperature. The following day, the sections were washed three times with distilled water and subsequently stained with hematoxylin to visualize nuclei, followed by differentiation and bluing. The sections were then stained with Biebrich scarlet solution, which rendered the cytoplasm and muscle fibers red. Following treatment with phosphomolybdic acid solution, the sections were counterstained with aniline blue, which stained collagen fibers and mucin blue. Finally, the sections were dehydrated, cleared in xylene, mounted, and scanned using a slide scanner.

### 2.9 Morphometrical analysis

The thicknesses of the granulation tissue and epithelium were measured perpendicular to the epithelial surface at five randomly selected sites in each sample, with adjacent measurements separated by at least 400 μm.

Collagen fiber organization, inflammatory cell density, and vascular density were evaluated using a semiquantitative scoring system ranging from 0 to 4, as previously described (Igrunkova et al., 2023), where 0 indicated the absence of the feature and four indicated its maximum expression. The presence of newly formed hair follicles was assessed using a binary scoring system: 0, no follicles in the central region of the wound; and 1, follicles present in the central region.

### 2.10 Immunohistochemical (IHC) staining

The sections were deparaffinized in xylene and rehydrated through a graded ethanol series (100%, 95%, 85%, and 75%). Antigen retrieval was then performed using EDTA-Tris alkaline buffer in a water bath. After cooling to room temperature, the sections were washed three times with phosphate-buffered saline (PBS) for 5 min per wash. To block endogenous peroxidase activity and reduce non-specific background staining, the sections were incubated in 3% hydrogen peroxide solution at room temperature for 15 min. After washing with PBS, the sections were blocked with QuickBlock blocking solution at room temperature for 30 min, washed three times with PBS (5 min per wash), and incubated overnight at 4 °C with primary antibodies (anti-IL-1β, anti-IL-6, and anti-TNF-α) diluted in antibody diluent in a humidified chamber. Serial sections from the same tissue block or sections from different mice within the same group were used for each primary antibody. The following day, the sections were washed three times with PBS, incubated with biotinylated secondary antibodies at room temperature for 30 min, washed again with PBS, and incubated with HRP-conjugated streptavidin at room temperature for 1–2 min. The reaction was developed using 3,3′-diaminobenzidine (DAB). The reaction was monitored under a microscope, and development was terminated by washing with distilled water once a brown precipitate appeared in the positive areas. The sections were subsequently counterstained with hematoxylin to visualize the nuclei, differentiated in acid alcohol, dehydrated through a graded ethanol series, cleared in xylene, and mounted with neutral resin. The stained sections were scanned and examined using a slide scanner.

### 2.11 Tyramide Signal Amplification (TSA)

After deparaffinization and rehydration, antigen retrieval was performed. The sections were then treated with 3% hydrogen peroxide at room temperature for 15 min and washed three times with PBS (5 min per wash). Non-specific background staining was reduced by blocking with QuickBlock blocking solution for 30 min. After removal of the blocking solution, the sections were incubated overnight at 4 °C with primary antibodies (CD206, CD68, and iNOS) diluted in antibody diluent. The following day, the sections were washed three times with PBS, incubated with the corresponding polymer HRP-conjugated rabbit secondary antibody, and processed according to the manufacturer’s tyramide signal amplification (TSA) protocol. Finally, the sections were mounted with an anti-fade mounting medium containing DAPI, imaged under a confocal microscope, and subjected to statistical analysis.

## 3 Results

### 3.1 The influence of pluronic F108 and sodium alginate on the luminescence spectrum of singlet oxygen upon photoexcitation of PS

To assess the singlet oxygen generation efficiency of the photosensitizers, the factors affecting the luminescence of ^1^O_2_ were investigated.

**FIGURE 1:**
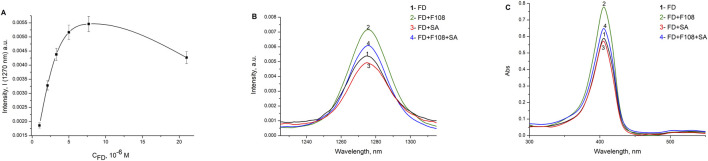
**(A)** Dependence of the singlet oxygen luminescence intensity at λ = 1270 nm on the concentration of FD in D_2_O at 293 K upon photoexcitation at λ = 405 nm. **(B)** Luminescence spectra of singlet oxygen (^1^O_2_) upon photoexcitation of FD (λ = 405 nm) in the absence and presence of BAC in D_2_O at 293 K. **(C)** Electronic absorption spectra of FD in the absence and presence of BAC in H_2_O at 293 K. Experimental conditions: [FD] = 7.7 × 10^−6^ M [F108] = 1 × 10^−4^ M and [SA] = 0.00125 wt%. Error bars represent the standard deviation for at least three independent experiments.

Figure 1A shows the variation in singlet oxygen luminescence intensity, I (λ = 1270 nm), as a function of FD concentration in aqueous solution.

The relationship is non-monotonic and exhibits a distinct maximum. The highest ^1^O_2_ luminescence intensity was observed at a photosensitizer concentration of 7.7 × 10^−6^ M. Further increases in FD concentration resulted in a decrease in I, most likely owing to aggregation of the photosensitizer in aqueous solution. This hypothesis is supported by a decrease in the optical density of the FD Soret band with increasing photosensitizer concentration (Kuryanova et al., 2023; Shershnev et al., 2025). Previous studies have shown that the rate of substrate photooxidation decreases with increasing photosensitizer concentration due to photosensitizer aggregation. As discussed above, amphiphilic polymers can be used to mitigate this aggregation-induced loss of activity.

Figure 1B shows the luminescence spectra of ^1^O_2_ generated by FD in the absence and presence of Pluronic F108 and SA. Addition of Pluronic F108 to an aqueous FD solution increased the ^1^O_2_ luminescence intensity (I) by approximately 1.3-fold compared with FD alone, using the model reaction of tryptophan photooxidation, that the singlet oxygen generation activity of porphyrin photosensitizers increases in the presence of amphiphilic polymers such as PVP, PVA, F127 and others (Aksenova et al., 2013; Gorokh et al., 2011; Kuryanova et al., 2023). The mechanism of this effect involved the formation of weakly bound intermolecular PS–AP complexes in the aqueous phase due to hydrophobic interactions and PS disaggregation. Amphiphilic polymers have also been shown to enhance the therapeutic efficacy of FD in tumor cell cultures and superficial tumor models (OOO Veta-Grand, 2009), as well as in the aPDT of purulent wounds in laboratory animals (Institute of Chemical Physics named after. N.N. Semenov RAS, 2006).

Gorokh et al. further reported that, under model conditions using the tryptophan photooxidation reaction, amphiphilic polymers increased the singlet oxygen generation activity of porphyrins by approximately 1.5–2-fold (Gorokh et al., 2011).

A decrease in the intensity of FD-generated ^1^O_2_ luminescence (I) was also observed in the presence of sodium alginate (10%), possibly due to interactions between FD molecules and the polyanionic SA. However, in the FD + F108 + SA ternary system I increase increased by approximately 24% relative to the FD + SA system. These findings suggest that Pluronic F108 reduces the interaction between FD and sodium alginate, thereby partially restoring the photosensitizing activity of FD. This interpretation is further supported by the corresponding absorption spectra (Figure 1C).

To quantitatively evaluate singlet oxygen generation by MB and its complexes with F108 and SA, the luminescence spectra of ^1^O_2_ were analyzed by calculating the integrated area (S) of the luminescence peak at λ = 1276 nm.

As shown in Table 3 the MB + F108 system exhibited an increase in S. In contrast, addition of SA to the MB solution decreased S by approximately 1.5-fold compared with MB alone. This decrease is apparently caused by electrostatic interactions between the cationic MB and the carboxyl groups of SA, which is consistent with our previous findings demonstrating reduced photocatalytic activity of MB in the tryptophan oxidation reaction in the presence of SA (Kuryanova et al., 2023). The addition of F108 to the MB + SA system slightly increases the S (lum 1276 nm) compared to that observed upon photoexcitation of the MB + SA system alone.

**TABLE 3:** The luminescence peak area of ^1^О_2_ singlet oxygen when using MB and MB-polymers in D_2_O at 293 K.

Sample	S_lum 1276 nm_,a.u
MB (1 × 10^−5^М)	0.188 ± 0.002
MB + F108 (1 × 10^-4^M)	0.197 ± 0.002
MB + SA (0.00125 wt%)	0.126 ± 0.003
MB + F108 + SA	0.147 ± 0.007

The results indicate that electrostatic interactions between photosensitizer molecules, which promote molecular aggregation, influence the efficiency of singlet oxygen generation (Kuryanova et al., 2023).

### 3.2 The influence of F108 and SA on the photostability of PS

**FIGURE 2:**
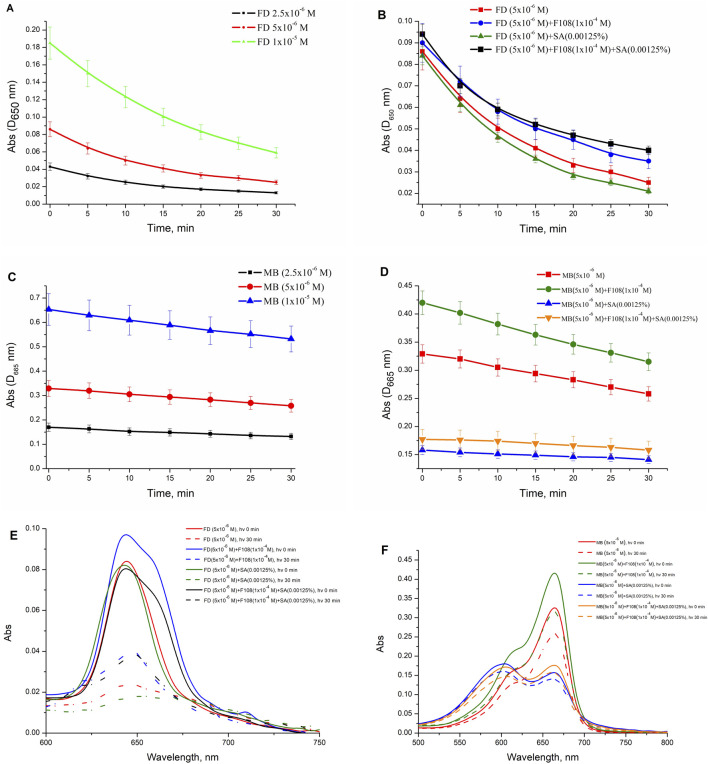
**(A,C)** Dependence of the optical density of FD (A, λ = 650 nm, ε = 17,000 M^-1^cm^-1^) and MB (C, λ = 665 nm, ε = 64,800 M^-1^cm^-1^) during irradiation at λ = 660 nm. **(B,D)** Dependence of the optical density of FD. **(B)** and MB. **(D)** on irradiation time in the absence and presence of polymers in H_2_O at 293 K. **(E,F)** Electronic absorption spectra of FD. **(E)** and MB. **(F)** in the absence and presence of F108 and SA before irradiation and after 30 min of irradiation in H_2_O at 293 K. Irradiation was performed using an LED source operating at λ = 660 nm. Error bars represent the standard deviation of at least three independent experiments.

Another important factor influencing the efficiency of PS in PDT is its photostability. Figures 2A–D present the photodegradation kinetics of FD and MB along, as well as their binary (PS + SA and PS + F108) and ternary (PS + F108 + SA) systems. Figures 2E,F shows the absorption spectra of FD and MB before irradiation and after 30 min of irradiation. As shown in Figure 2A, irradiation at λ = 660 nm with a power density of 1400 W/m^2^ resulted in up to 70% degradation of Fotoditazin within 30 min, regardless of its initial concentration. According to Bagrova et al., the relatively low photostability of porphyrin photosensitizers such as Dimegin and Fotoditazin arises from the susceptibility of their highly conjugated porphyrin macrocycles to oxidative attack by ROS generated during photoexcitation (Bagrov et al., 2018).

According to Rotomskis et al. (1997), such interactions can lead to opening of the porphyrin macrocycle, resulting in the appearance of a new absorption band in the UV region of the porphyrin spectrum (Rotomskis et al., 1997). In the present study, no such changes in the EAS of FD were observed upon irradiation at λ = 660 nm, suggesting that opening of the aromatic macrocycle most likely did not occur. This conclusion is supported by the absorption spectra of FD recorded before and after 30 min of irradiation (Supplementary Figure S3).

Methylene blue is generally considered a photostable dye (Khan et al., 2022; Tayade et al., 2009). Indeed, as shown in Figure 2C, MB underwent only approximately 20% photodegradation after 30 min of irradiation. It is reasonable to assume that efficiency of singlet oxygen generation by a photosensitizer (i.e., its singlet oxygen quantum yield) and its photostability are interrelated (Ferreira et al., 2008; Ferreira et al., 2009). In general, photosensitizers with higher singlet oxygen quantum yields tend to exhibit lower photostability. Both Pluronic F108 and SA influence the photostability of the photosensitizer under irradiation at λ = 660 nm (Figures 2B,D). In the presence of Pluronic F108, the photostability of FD increases, with photodegradation reaching only 61% over the 30-min observation period (Figure 2B, blue curve). This effect can be attributed to the “protective” role of amphiphilic polymer. Pluronic solubilizes Fotoditazin, and incorporates it into micellar structures, thereby reducing interactions between the photosensitizer and ROS and slowing photodegradation while preserving the ability of FD to generate ^1^O_2_. These findings are also reflected in the corresponding change in the FD absorption spectra (Figure 2E). Over the same period, the photostability of methylene blue in the MB + F108 system remained virtually unchanged (Figure 2D, green curve). This is possibly due to the absence of a macrocyclic double bond system in the MB structure, which is easily decomposed by ROS. Accordingly, MB is more resistant to interaction between MB and the anionic polysaccharide SA leads to a decrease in optical density due to the aggregation of dye molecules, making it even less susceptible to photodegradation (Figure 2D, blue curve).

The photostability of MB in the binary MB + SA and ternary MB + F108 + SA systems was essentially identical, indicating that, under the present experimental conditions, Pluronic F108 was unable to prevent the electrostatic interactions between MB and SA. As noted above, ionic interaction in the MB and SA system leads to the aggregation of PS (in particular to dimerization of the dye), which is confirmed by the absorption spectra of MB (Figure 2F). We have previously shown that the resulting MB + SA system leads to a decrease in the photodynamic activity of MB (a decrease in the fluorescence intensity and optical density of the main absorption band of MB at λ = 660 nm was observed) (Kuryanova et al., 2023). However, the additional introduction of amphiphilic polymers at concentrations above 10^−4^M (especially PVP) into the MB + SA system can restore both the photosensitizing activity of methylene blue and, accordingly, the spectral characteristics of the dye (EAS, fluorescence spectrum) (Kuryanova et al., 2023).

Adding SA to an aqueous FD solution does not reduce the rate of Fotoditazin photodegradation (Figure 2B, green curve). FD becomes most photostable in the FD + F108 system (Figure 2B, blue curve). The highest photostability of FD is observed in the FD + F108 + SA system (Figure 2B, black curve). This effect is most likely due to the interaction of FD with Pluronic F108 within its micelles, where Pluronic protects FD from light exposure, thereby slowing the photodegradation process.

### 3.3 Macroscopic assessment of the clinical picture of the wound condition

**FIGURE 3:**
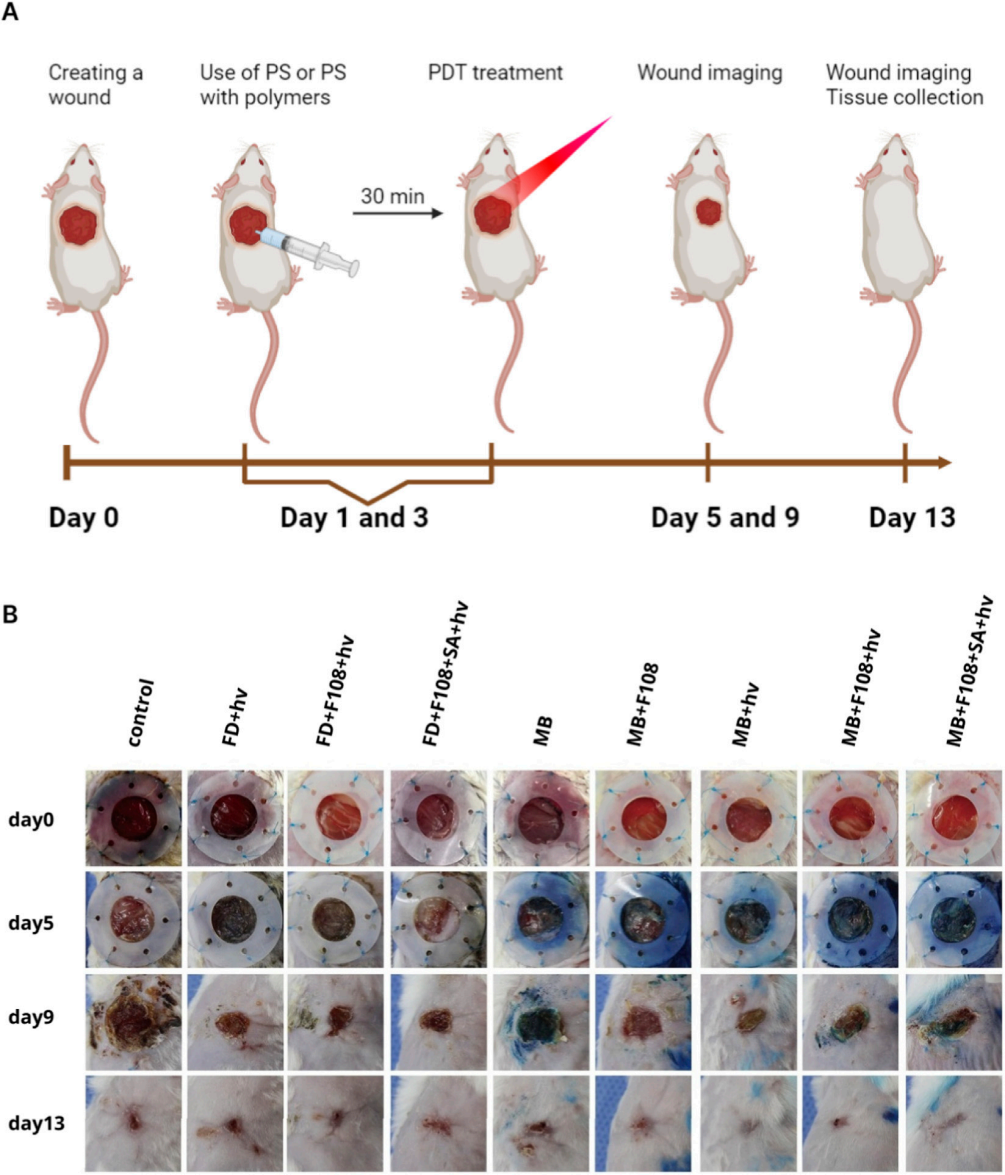
**(A)** Schematic illustration of the experiment schedule for PDT treatment. **(B)** Representative images of wound healing in mice under different treatment conditions. Photographic timeline of wound healing in mice over 13 days, comparing nine treatment groups. Images were taken on days 0, 5, 9, and 13 after wound infliction.

In the early postoperative period, the clinical appearance of the wounds was similar across all study groups. During the first 24 h after surgery, the wounds exhibited moderate moisture, minimal exudation, and a mild perifocal response. The wound bed was covered by a thin whitish fibrin layer. The experiment schedule is shown in Figure 3A.

The dynamics of wound healing are presented in Figure 3B, which shows representative photographs of the wounds in all study groups at key experimental time points. All groups subjected to photodynamic therapy (PDT) demonstrated a statistically significant acceleration of wound healing compared with both the untreated control group and the non-irradiated groups. Among the PDT-treated groups, the MB + F108 + SA + hv system (Group IX) demonstrated the greatest therapeutic efficacy and the fastest rate of wound closure (Figure 3B).

To objectively evaluate tissue regeneration, a planimetric analysis of wound area was performed. In Group IX, wound closure was nearly complete by day 13. It should be noted that during the first 5 days after wound creation, an insulating ring secured with six sutures was used to prevent wound edge contraction and maintain a standardized wound cavity. This approach enabled the assessment of regenerative tissue filling within the defect, rather than the effects of peripheral contraction.

**FIGURE 4:**
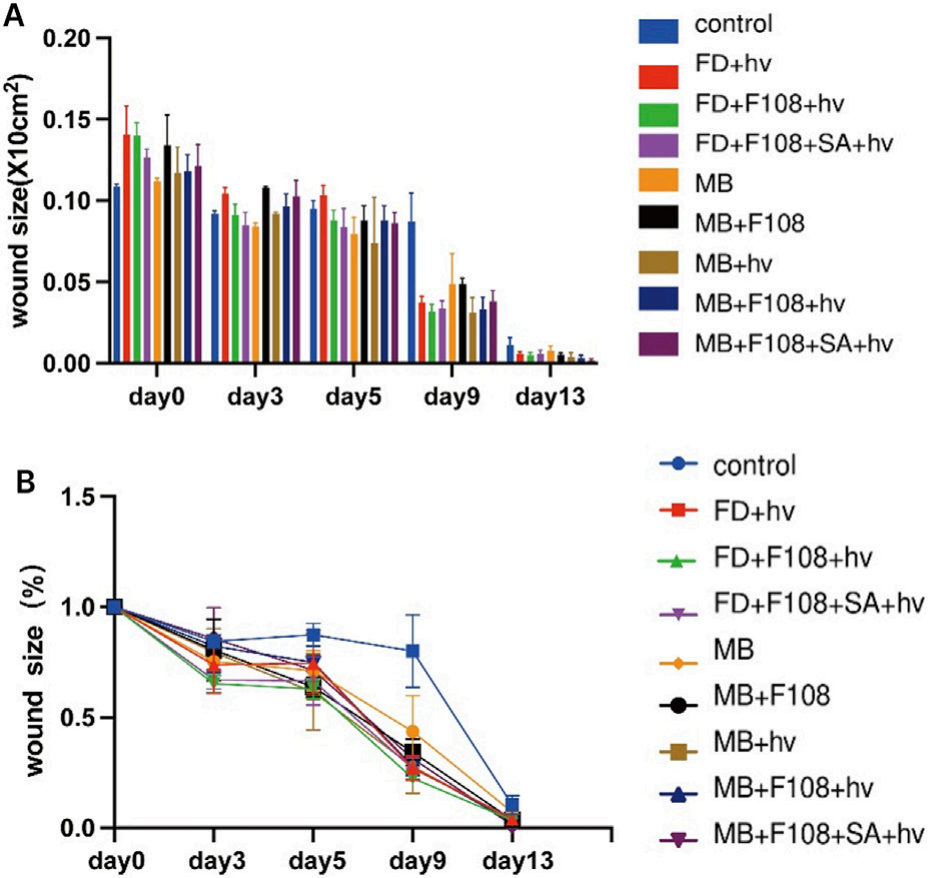
**(A)** Quantitative assessment of wound healing progress under different treatment conditions. Histogram representing the wound area from day 0 to day 13 for each treatment group. **(B)** Graph of the wound closure rate over time. Data are presented as mean ± standard deviation. ****p < 0.0001 compared to the control group (two-way ANOVA with Tukey’s post-hoc test). n = 4 in each group.

Figures 4A,B presents a quantitative evaluation of wound healing. The histogram (Figure 4A) illustrates the temporal changes in wound area from day 0 to day 13 for each group, while the line graph (Figure 4B) depicts the rate of wound closure. In all aPDT-treated groups, near-complete closure of the wound defect was observed by day 13, with the greatest reduction in wound area recorded in Group IX. In the control group, the wound area did not change significantly during the first 10 days of the experiment. No significant changes in wound size were observed in any group during the first 5 days, most likely due to the PDT procedures performed on days 1 and 3. By day 3, the formation of granulation tissue was visible, although in some cases a persistent fibrin-leukocyte layer remained.

Thus, with respect to the rate of wound closure, Group IX exhibited the best result. However, to assess not only the rate but also the quality of the newly formed tissue, a detailed histological analysis was performed on day 13.

### 3.4 Results of histological examination

**FIGURE 5:**
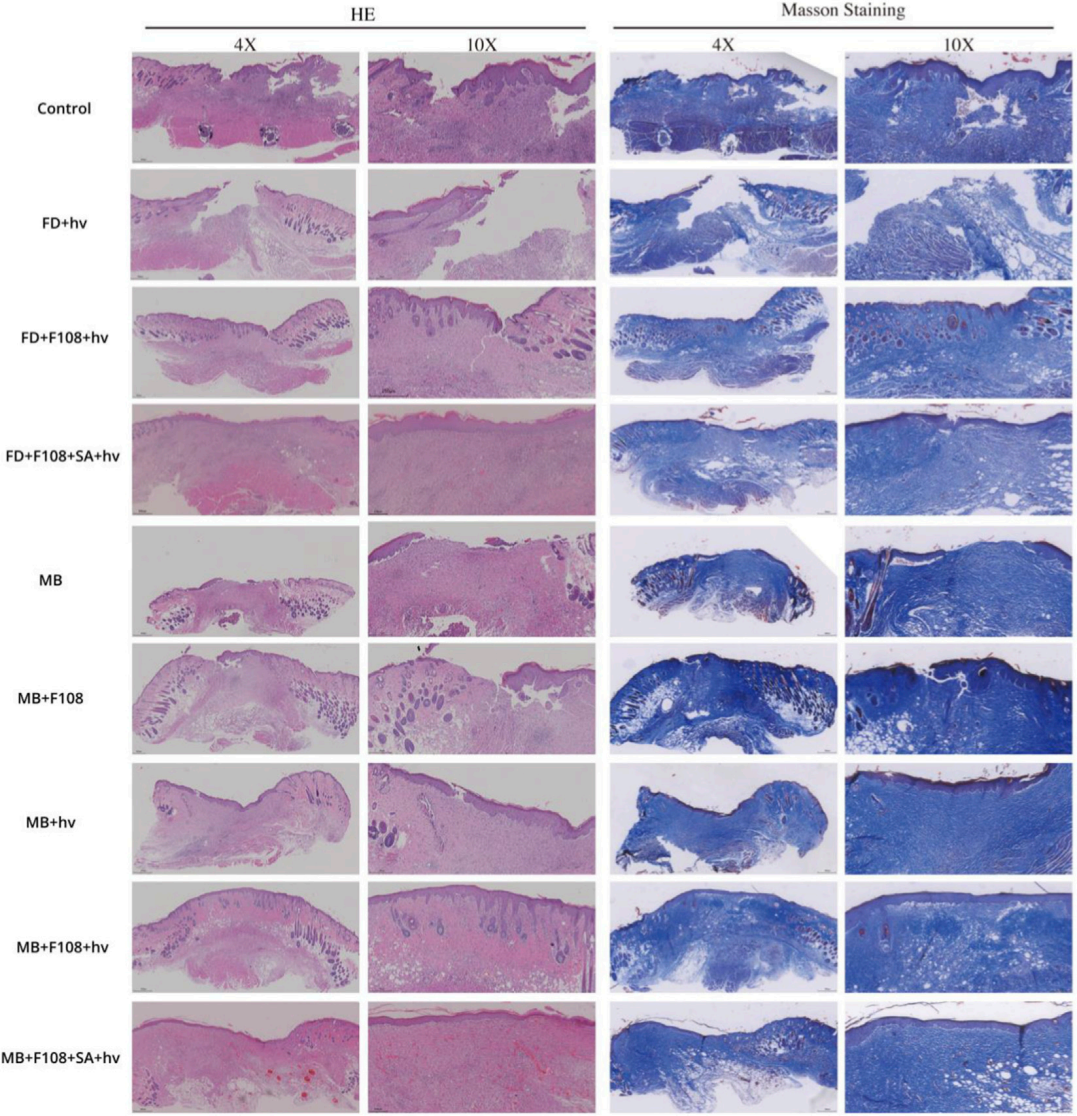
Representative histological images of the central region of the wound bed in the different study groups. Sections were stained with hematoxylin–eosin (magnification ×4 and ×10) and Masson’s trichrome (magnification ×4 and ×10).

Histological analysis of the tissues formed within the wound defect by the 13th day after surgery, following two sessions of photodynamic therapy, revealed that all groups exhibited a generally similar three-layered tissue organization: a superficial fibrin-leukocyte layer (exudate), a middle granulation tissue layer, and a deep adipose tissue layer. However, the morphological features of each layer varied significantly among groups, apparently reflecting different photobiological effects (Figure 5). To evaluate morphological findings of inflammation and regeneration, we used a 0 to four score system (0—absence of the findings, 4—maximum intensity of the findings) (Igrunkova et al., 2023). To estimate the number of new follicles, we used a binary system: 0—no follicles in the central zone, 1—follicles present in the central zone.

• Group I (no treatment). Almost complete epithelialization was observed (epithelial thickness, 130 μm; granulation tissue thickness, 1100 μm; maturity score, 1; vascular density score, 1); however, the dermis exhibited dense infiltration by immune cells, predominantly neutrophils, consistent with acute septic inflammation extending through the full thickness of the skin (∼1 mm) (inflammatory cell density score, 4) (Figure 5).

• Control groups II and V (FD + hv, MB) exhibited similar histological features. By day 13, focal infiltration of immune cells persisted (inflammatory cell density indicator one and two respectively). The extracellular matrix showed evidence of reorganization, characterized by individual thick collagen fiber bundles lacking the typical reticular architecture. The collagen fibers were thin and immature, as indicated by pale Masson’s trichrome staining, and the granulation tissue remained loose (granulation tissue thickness 700 μm and 500 μm; maturity scores of one and 2; vascular density scores 0 and 2, respectively). These features are indicative of a prolonged inflammatory process, likely related to the septic nature of the wound (epithelial thickness 210 μm and 100 μm, number of regenerated hair follicles score for both is 0).

• Control group III (FD-F108+hv). Animals in this group exhibited a more pronounced regenerative response compared with the preceding control groups, as evidenced by more extensive and mature granulation tissue (granulation tissue thickness, 1000 μm; maturity score, 3), the formation of a dense extracellular matrix (although without a reticular structure), increased cellularity, and early signs of skin appendage regeneration. A small focal infiltration of immune cells was present (inflammatory cell density score, 1), which is characteristic of the healing phase. The intensity of inflammation and signs of microcirculation impairment (vascular density score, 1) did not differ from other control groups (epithelial thickness, 140 μm, number of regenerated hair follicles score, 1).

• Groups VI and VII (MB + F108, MB + hv). Both groups showed signs of active regeneration without pronounced inflammatory (inflammatory cell density score, 1); however, the regenerative patterns differed (Group VI: epithelial thickness 90 μm; granulation tissue thickness 700 μm; maturity score, 3; vascular density score, 3; and number of regenerated hair follicles score, 0. Group VII epithelial thickness 170 μm; granulation tissue thickness 600 μm; maturity score, 2; vascular density score, 1; and number of regenerated hair follicles score, 0).

• Group VIII (MB + F108 + hv). The histological appearance differed markedly from all previous observations. A qualitative restoration of the dermis was evident, including regeneration of skin appendages and reestablishment of the reticular architecture of the extracellular matrix, indicating true tissue regeneration rather than simple scar formation. In the central region of the sample, newly formed skin appendages were observed (epithelial thickness 110 μm; inflammatory cell density score, 0), while the deeper dermal layers exhibited clear signs of active regeneration, including blood-filled capillaries and vertically oriented vascular loops (granulation tissue thickness 500 μm; maturity score, 4, vascular density score, 3, number of regenerated hair follicles score, 1). The quality of the newly formed collagen was confirmed by its intense blue staining with Masson’s trichrome.

• Group IV (FD + F108 + SA + hv). Complete epithelialization of the defect was observed (epithelial thickness 70 μm). The newly formed stratified keratinizing epithelium was continuous, with thickness and layering comparable to normal epidermis; however, skin appendages (hair follicles) were absent (number of regenerated hair follicles score, 0). Within the depth of the defect (∼2 mm), fibrotic granulation tissue infiltrated by immune cells (inflammatory cell density score, 2), and characterized by a loose extracellular matrix was identified. Masson’s trichrome staining revealed an almost complete absence of collagen in the central region of the defect, with only sparse thin fibers present between clusters of immune cells. Immediately beneath the epithelium, a thin layer of mature granulation tissue was observed (granulation tissue thickness 100 μm; maturity score, 2, vascular density score, 3), consisting of spindle-shaped fibroblasts oriented parallel to the epithelium. The adjacent zone of infiltration indicates that the inflammatory process limited fibroblast migration and the transition from the inflammatory to the proliferative phase of healing.

• Group IX (MB + F108 + SA + hv). The histological picture corresponded to the proliferative phase of healing and demonstrated partial reorganization of the dermal matrix. Regeneration of hair follicles was observed (number of regenerated hair follicles score, 1), which was absent in the corresponding FD-based group (Group IV). Inflammation was localized (inflammatory cell density score, 0), and a thick layer of dense connective tissue extended beneath the epithelium, reflecting active fibroblast migration from the wound edges (epithelial thickness, 90 μm; granulation tissue thickness, 400 μm; maturity score, 3; vascular density score, 4).

In the FD groups, a marked thickening of the epithelial layer is observed, which is likely related to the formation of scar tissue due to active regeneration. In groups IV, VI, and IX, epithelial thickness is within normal limits. For groups I-IV high granulation tissue thickness was observed, likely related to tissue edema, which is clearly visible during visual assessment of sample sections. This indicator is two times lower in groups VIII and IX. Based on a combination of parameters, groups VIII and IX demonstrated high granulation tissue maturity and minimal inflammation, in contrast to the FD groups with similar polymer compositions and a high degree of vascular neoplasms.

To further investigate the effects of aPDT on vital internal organs, the visceral organs of all experimental mouse groups were examined, and the results showed no obvious visceral organ toxicity (Supplementary Figure S3).

### 3.5 Composite photosensitizers suppress pro-inflammatory cytokines

**FIGURE 6:**
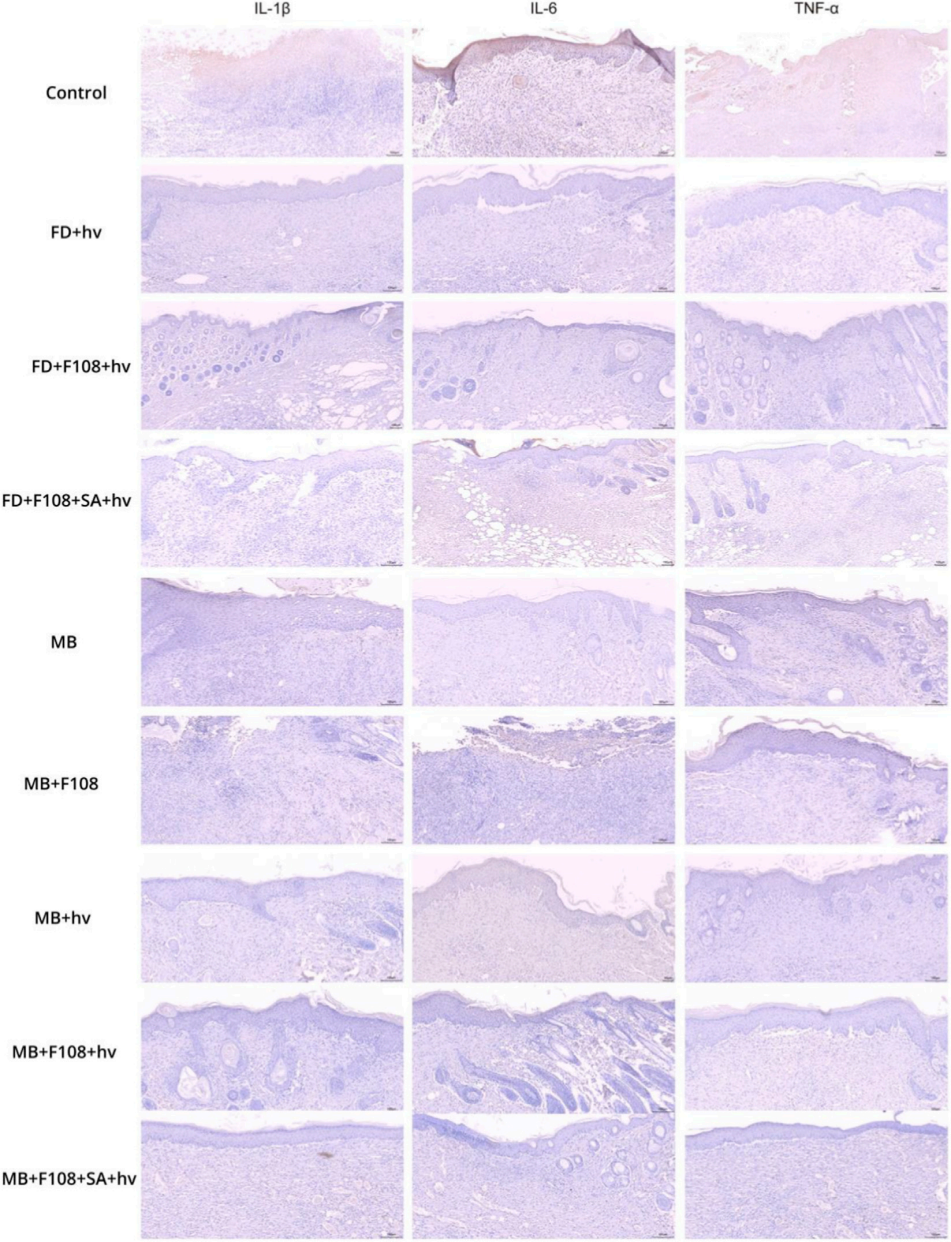
Representative IHC images of the central region of the wound bed in the different study groups. Sections were stained with IL-1β, IL-6, TNF-α (magnification ×20).

To further elucidate the effects of different treatments on the wound microenvironment, immunohistochemical (IHC) staining of key inflammatory cytokines was performed. IHC analysis revealed significant differences in pro-inflammatory cytokine expression between the control group and the aPDT-treated groups. In control group wound tissue, IL-1beta, IL-6, and TNF-alpha exhibited strong positive expression. Conversely, all aPDT-treated groups showed downregulation of pro-inflammatory cytokine expression. The MB + F108 + SA + hν and FD + F108 + SA + hν groups demonstrated the most pronounced inhibitory effects, indicating that effective pathogen clearance via aPDT rapidly downregulates pro-inflammatory factors, thereby facilitating the transition to subsequent tissue repair stages (Figure 6).

### 3.6 aPDT modulates macrophage polarization from a pro-inflammatory M1 to a pro-repair M2 phenotype

**FIGURE 7:**
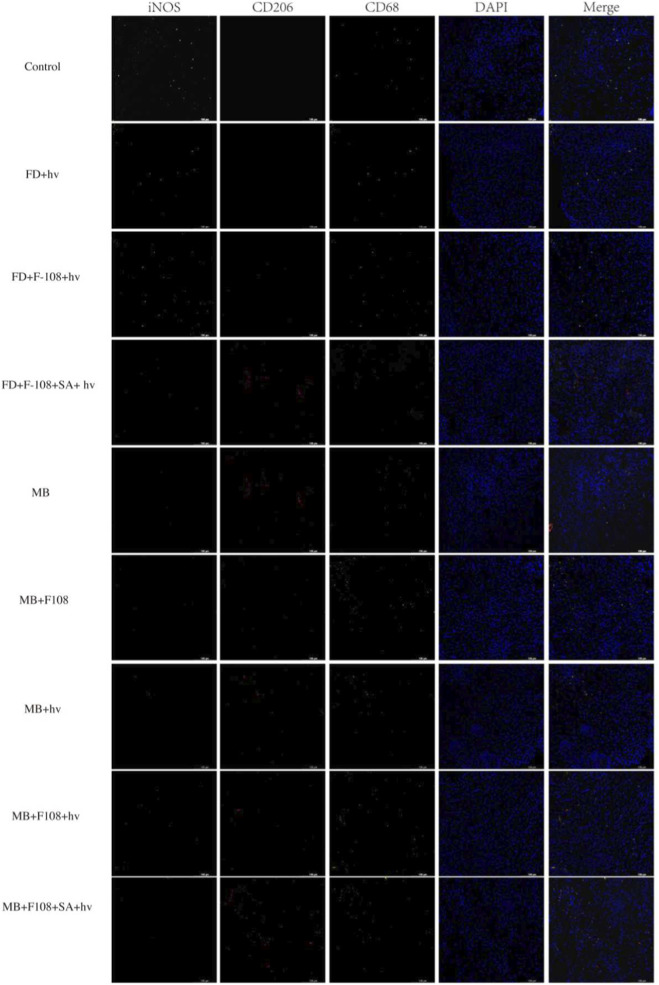
Representative IF images of the central region of the wound bed in the different study groups. Sections were stained with iNOS, CD206, CD68 (magnification ×20).

To investigate the effects of different treatment regimens on macrophage polarization, tyramide signal amplification (TSA) staining was used to analyze the distribution and proportions of total macrophages (CD68^+^), M1 macrophages (iNOS^+^), and M2 macrophages (CD206^+^). Fluorescent staining revealed extensive infiltration of CD68^+^ macrophages in the wound tissue of the control group. In contrast, the number of CD68^+^ cells decreased in all aPDT-treated groups, indicating that effective pathogen clearance reduced chemotactic signals for macrophage recruitment. In the MB + F108 + SA + hν and FD + F108 + SA + hν groups, M2 macrophage infiltration predominated. In contrast, control wound tissue exhibited a strong fluorescence signal dominated by iNOS^+^ M1 macrophages, while CD206^+^ M2 macrophages were scarce, resulting in a severe M1/M2 imbalance. This imbalance explains why control group wounds remained persistently inflamed and failed to progress into the repair phase (Figure 7).

## 4 Discussion

The morphological data indicate that wound healing is a complex, dynamic, and multifactorial process involving coordinated cellular and molecular events. The system with MB proved to be the most effective. MB appears to suppress septic inflammation (unlike photodynamic therapy alone), and in the last two groups (VIII - MB + F108 + hv and IX - MB + F108 + SA + hv), it provided a near-complete qualitative restoration of the skin’s microstructure. These findings demonstrate potential application prospects worthy of further exploration.

The experimental design employed a murine full-thickness excisional infected wound splinting model, a widely adopted preclinical methodology in which silicone splint rings prevent wound edge contraction, thereby directing healing primarily through granulation tissue formation and re-epithelialization—processes that simulate, to a certain extent, some of the mechanisms of human cutaneous wound repair (Rhea and Dunnwald, 2020; Wang et al., 2013). The use of a *S. aureus*-infected wound model further enhanced clinical relevance, as polymicrobial colonization represents the most prevalent complication in human chronic wounds (Wang et al., 2013).

The findings can be interpreted through the classical four-phase framework of cutaneous repair: hemostasis, inflammation, proliferation, and remodeling (Gurtner et al., 2008; Peña and Martin, 2024; Reinke and Sorg, 2012). Although inflammation is essential for preventing infection, its persistence establishes a pathological positive feedback loop that impedes transition to the proliferative phase (Gurtner et al., 2008). The proliferative phase encompasses re-epithelialization, angiogenesis, and granulation tissue formation, coordinately regulated by keratinocytes, fibroblasts, and endothelial cells (Peña and Martin, 2024). The remodeling phase involves collagen maturation and restoration of reticular dermal architecture, distinguishing true regeneration from fibrotic scar formation (Gurtner et al., 2008; Reinke and Sorg, 2012). Our histological findings in Groups VIII and IX—characterized by complete epithelialization, restoration of reticular collagen architecture, and *de novo* hair follicle formation—suggest that MB-based antimicrobial photodynamic therapy containing F108 and SA may promote features associated with regenerative rather than purely reparative healing (Gurtner et al., 2008).

The presented results are in good agreement with the known mechanisms of action of methylene blue during PDT at all stages of wound healing (Makarov et al., 2024; Minagawa et al., 2023; Rosique et al., 2017). During photodynamic therapy, methylene blue acts as a cyclic electron acceptor, integrating into the mitochondrial respiratory chain and enhancing electron transfer and ATP (energy) synthesis. This creates a favorable metabolic environment for tissue regeneration (Makarov et al., 2024; Nedu et al., 2020). Additionally, methylene blue mitigates oxidative stress in wounds, thereby promoting a more rapid transition from the inflammatory phase to the proliferative phase of healing (OOO Veta-Grand, 2009).

The proliferative phase is particularly critical for the outcomes observed in Groups VI–IX, as this stage determines the quality and durability of tissue repair. During this phase, the MB-assisted photodynamic therapy (MB-PDT) is known to upregulate vascular endothelial growth factor (VEGF) expression, thereby promoting angiogenesis and revascularization of the wound bed (Minagawa et al., 2023). This explains the active capillary formation observed in Groups VII, VIII, and IX. Simultaneously, MB-PDT significantly enhances fibroblast proliferation and the synthesis of type I collagen—the principal structural component of the extracellular matrix—thereby promoting restoration of dermal integrity (Karamanos et al., 2021; Makarov et al., 2024; Minagawa et al., 2023; Nedu et al., 2020; Rosique et al., 2017). Apparently, this mechanism is implemented in the groups with MB-PDT in the absence and presence of the polymer and SA (Groups VII, VIII, IX), where active collagen synthesis was observed. In contrast, collagen maturation was virtually absent in Group IV (FD + F108 + SA + hv).

This study demonstrated the important role of the polymer and polysaccharide components in the photosensitizer complex. Incorporation of the polymeric components F108 and SA into the system moderately improved the photosensitizer photostability by approximately 10%–15%. Methylene blue (MB) itself exhibits high photostability (the degree of photodegradation is 22%, under experimental conditions); however, in the resulting ternary system MB + F108 + SA, the degree of photodegradation rate decreased to 11%. This enhanced photostability has clinical implications, as it extends the effective therapeutic window during irradiation.

The polymer F108 promotes disaggregation of the cationic dye, thereby enhancing the photodynamic efficacy of MB in PDT. This effect was most pronounced in Group VIII, where features suggestive of advanced tissue regeneration were observed—including partial restoration of skin architecture. The presence of the SA in combination with MB and F108 (Group IX) during irradiation also showed a biological effect, particularly stimulating the appearance of hair follicles, which was not observed in its absence (Group VIII). However, matrix remodeling progression in Group IX was slower than in Group VIII, most likely because electrostatic interactions between cationic MB molecules and anionic alginate polymers partially attenuated the photosensitizing efficiency of MB in generation of singlet oxygen (^1^O_2_). Our previous work has demonstrated that amphiphilic polymers such as F108 can inhibit the ionic interaction of methylene blue with sodium alginate, thereby preserving the photocatalytic activity of MB (Chin et al., 2008).

The immunomodulatory effects of aPDT observed in this study—specifically, suppression of pro-inflammatory cytokines and macrophage phenotypic switching from M1 to M2—extend beyond mere pathogen clearance (Fred, 2024; Rodrigues et al., 2019). Macrophage polarization between M1 (pro-inflammatory, iNOS^+^) and M2 (pro-reparative, CD206^+^) phenotypes constitutes a critical regulatory node determining tissue repair trajectory (Fred, 2024; Rodrigues et al., 2019). Our TSA staining results demonstrated that control group tissues exhibited severe M1/M2 imbalance dominated by iNOS^+^ M1 macrophages, whereas MB + F108 + SA + hν treatment induced an apparent trend toward CD206^+^ M2 predominance. The mechanistic basis likely involves the combined action of pathogen clearance (eliminating pathogen-associated molecular patterns [PAMPs] that sustain M1 activation) and ROS-dependent regulation of redox-sensitive transcription factors (Khorsandi et al., 2022; Sahu et al., 2015).

Thus, the results are highly promising in promoting regenerative rather than fibrotic processes during photodynamic therapy, highlighting their potential for enhancing wound healing outcomes. The use of methylene blue in combination with F108 and SA polymers under light irradiation (Groups VIII - MB + F108 + hv and IX - MB + F108 + SA + hv) represents an effective strategy for targeted regulation of wound healing phases, promoting not only rapid wound closure but also qualitative restoration of normal skin architectonics.

## 5 Conclusion

Thus, the results indicate that while FD exhibits a high quantum yield of singlet oxygen generation, it is also highly susceptible to photodecomposition. Under the experimental conditions, up to 70% of FD undergoes degradation (with destruction of its chemical structure) within 30 min. The incorporation of polymer components — F108 and SA — into the system enhances photosensitizing activity and moderately improves the photostability of FD (by ∼10–15%). MB, in contrast, demonstrates high photostability, which further increases in the ternary MB + F108 + SA system. These differences in photostability between the photosensitizers, arising from the specific features of their chemical structures, should be taken into account when selecting a treatment strategy for model wounds in PDT.

Such optimization of physicochemical properties observed *in vitro* ultimately translated into significant biological benefits in the treatment of model wounds in animals using PDT. Macroscopically, two combination treatment groups (Groups IV - FD + F108 + SA + hv and IX - MB + F108 + SA + hv) exhibited the fastest wound healing rate, with wounds essentially closed by day 9. Moreover, MB suppressed septic inflammation (unlike FD), and in Groups VIII (MB + F108 + hv) and IX (MB + F108 + SA + hv), it even ensured almost complete qualitative restoration of skin microstructure. These results suggest that MB-based combination therapy may serve as a potential candidate worthy of further validation.

At the microscopic level, Masson’s trichrome results jointly confirmed that complex treatment, especially with MB, not only promoted neo-epithelialization and mature granulation tissue formation but, more importantly, induced high-quality tissue remodeling, characterized by dense and orderly deposition of collagen fibers.

In summary, Pluronic F108 improves photosensitizer photostability and singlet oxygen production by reducing aggregation and providing physical protection, while sodium alginate creates a favorable wound healing microenvironment. Together, these polymers enable aPDT to modulate inflammatory and immune responses, leading to rapid and high-quality tissue regeneration. Group IX (MB + F108 + SA + hv) was particularly effective, indicating that MB-based formulations are preferable for infected wound treatment due to their superior photostability and immunomodulatory properties. This work provides a theoretical and experimental basis for developing efficient polymer–photosensitizer systems for wound therapy.

## Data Availability

The raw data supporting the conclusions of this article will be made available by the authors, without undue reservation.
