# Supplementary material for: Features of the wound healing process in mice during photodynamic therapy with fotoditazin and methylene blue in the presence of amphiphilic polymers and sodium alginate
Source: Front Chem. 2026 Jul 24;14:1852721. doi: 10.3389/fchem.2026.1852721 (PMC13447495; doi:10.3389/fchem.2026.1852721)
Supplement: Supplementary file 1 [file DataSheet1.docx]

Supplementary Material

**Supplementary Figure S1.** Structure of photosensitizers and polymers.

**Supplementary Figure S2.** Electronic absorption spectrum of FD at 0min of irradiation and at 30 min of irradiation in H_2_O at 293 K.

**Supplementary Figure S3.** Histopathological assessment of systemic toxicity in major organs following photodynamic therapy (magnification ×40).
